# Changes in Plasma Membrane Surface Potential of PC12 Cells as Measured by Kelvin Probe Force Microscopy

**DOI:** 10.1371/journal.pone.0033849

**Published:** 2012-04-10

**Authors:** Chia-Chang Tsai, Hui-Hsing Hung, Chien-Pang Liu, Yit-Tsong Chen, Chien-Yuan Pan

**Affiliations:** 1 Institute of Atomic and Molecular Sciences, Academia Sinica, Taipei, Taiwan; 2 Institute of Zoology, National Taiwan University, Taipei, Taiwan; 3 Department of Chemistry, National Taiwan University, Taipei, Taiwan; 4 Department of Life Science, National Taiwan University, Taipei, Taiwan; Emory University/Georgia Insititute of Technology, United States of America

## Abstract

The plasma membrane of a cell not only works as a physical barrier but also mediates the signal relay between the extracellular milieu and the cell interior. Various stimulants may cause the redistribution of molecules, like lipids, proteins, and polysaccharides, on the plasma membrane and change the surface potential (Φ_s_). In this study, the Φ_s_s of PC12 cell plasma membranes were measured by atomic force microscopy in Kelvin probe mode (KPFM). The skewness values of the Φ_s_s distribution histogram were found to be mostly negative, and the incorporation of negatively charged phosphatidylserine shifted the average skewness values to positive. After being treated with H_2_O_2_, dopamine, or Zn^2+^, phosphatidylserine was found to be translocated to the membrane outer leaflet and the averaged skewness values were changed to positive values. These results demonstrated that KPFM can be used to monitor cell physiology status in response to various stimulants with high spatial resolution.

## Introduction

The plasma membrane constitutes the boundary of a cell. This membrane is responsible for interactions between the extracellular environment and the cell interior. The basic structure of the membrane is a lipid bilayer, which is impermeable to most water-soluble molecules and forms a stable barrier between the inside and outside compartments [1], [2]. Various ion channels and transporters in the membrane are responsible for ionic homeostasis, and numerous proteins with polysaccharide modifications are involved in signal transduction and cell-cell recognition.

One of the main differences among phospholipids is the charges carried by the polar groups, which are exposed on the surface of the membrane. The distributions of phospholipids are asymmetric at both sides of the lipid bilayer. The inner and outer leaflets of the membrane are mainly composed of uncharged sphingomyelin, phosphatidylethanolamine, and phosphatidylcholine (PC), but in differing proportions. Under normal physiological conditions the negatively charged phosphatidylserine (PS) and phosphatidylinositol are localized at the inner leaflet. Disturbance of the asymmetric distribution, especially the appearance of PS at the outer leaflet, has been recognized as a cell death signal [3].

The membrane proteins contain charged amino acid residues and are usually modified by polysaccharides, in a process called glycosylation, at the extracellular domains. The glycosylation modifications form a glycocalyx layer outside the membrane and are important for protection and signal recognition [4], [5]. Some of these monosaccharides are negatively charged, such as sialic acid and heparin sulfate. Removing sialic acid from injured ganglion neurons hyperpolarizes the membrane potential [6]. Moreover, changing the glycosylation level influences cell differentiation, cancer development, and apoptosis [6], [7], [8], [9]. Therefore, a change in the surface charge is an important indicator of cellular activities.

Atomic force microscopy (AFM) is a powerful tool in visualizing [10], [11] and manipulating [12], [13] biological structures with nanometer resolution. Previous research has employed AFM to characterize the electrostatic properties of artificial plasma membranes composed of PS and PC [14]. Although AFM measures the surface charge at high resolution, it is sensitive to topographic artifacts and is not appropriate for measuring native plasma membrane with its wide fluctuations in height [15]. AFM can also be applied in Kelvin probe mode (KPFM) to investigate the electrical properties of materials, including biomolecules. KPFM measures the potential difference between two surfaces at close proximity and is less influenced by fluctuations in membrane height [15], [16], [17]. Therefore, when the tip of AFM is in close contact with the plasma membrane, the surface potential (Φ_s_) caused by the charged molecules can be revealed at high resolution.

The current study used KPFM to measure the changes in Φ_s_ of PC12 cells after they had been treated with various stimulants. Our results showed that the Φ_s_ at the membrane surface was not homogeneous. The distribution histograms of the Φ_s_s from each scanned membrane patch showed different asymmetry profiles. The skewness values, which represent the asymmetry patterns of distribution histograms, shifted positively in PC12 cells after they had been treated with reactive oxygen species (ROS). Changing the membrane outer leaflet composition by incorporating charged PS micelles also changed the Φ_s_s distribution profile. Fluorescence-tagged annexin V, which binds specifically to PS, was used to stain the ROS-treated PC12 cells and identify the increased PS exposure on the membrane outer leaflet. These results suggested that the Φ_s_s of the outer membrane surface as measured by KPFM can be applied to analyze the physiological conditions of cells in response to various stimulants.

## Methods

### Chemicals

Dulbecco's modified Eagle's medium (DMEM) and all other reagents for cell culture were obtained from Invitrogen Inc (Carlsbad, CA). The PC (L-α-lecithin, egg yolk, 524617) was purchased from EMD Chemicals Inc (Darmstadt, Germany), and the PS (L-α-phosphatidylserine, bovine brain, Fluka 79406) was purchased from Sigma-Aldrich (USA). All other chemicals were reagent grade and were purchased from Sigma-Aldrich, unless otherwise indicated.

### Cell preparation

The PC12 cells, derived from pheochromocytoma of the rat adrenal medulla, were generously provided by Dr. Lung-Sen Kao [18], [19]. The cells were cultured in DMEM containing 10% horse serum and 5% fetal bovine serum, and were then incubated at 37°C in an atmosphere containing 10% CO_2_. The medium was changed every two days. The cells grew to 60% confluence and were then treated with 100 µM H_2_O_2_, 1.25 mM dopamine, or 1 mM ZnCl_2_. Treatment was continued for 18 h in an incubator and the cells were then fixed with 3.7% paraformaldehyde in phosphate buffered saline (PBS) (137 mM NaCl, 2.7 mM KCl, 10 mM Na_2_HPO_4_, and 2 mM KH_2_PO_4_; pH 7.4) for 30 min. After being washed three times with PBS, the fixed cells were washed with deionized water to remove the salts, and were then dried by N_2_ gas flow in preparation for KPFM scanning.

### Micelle incorporation

Phospholipids were dissolved in a chloroform-methanol solution (3∶2) at a concentration of 1 mg/ml. The surface tension of PS or PC in PBS buffer was measured using a tensiometer to characterize the critical micelle concentration (CMC)(Supporting Information Figure S1). The surface tension was measured by pendant drop profile analysis tensiometry (PAT-2P, Sinterface Technologies, Germany). The temperature of the measuring glass cell (volume V = 20 ml) was kept constant at 25°C and the sample cell was closed using a lid with an immersed capillary tip, in which the drop of a certain volume of solution was formed. Each drop of sample solution was formed at the tip of the steel capillary with a manual injection to make a drop in equilibrium before reckoning. The surface tension of the pendant drop was obtained by viewing the shape with a low-magnification lens (2×) and the dimensions of the pendant drop were determined from the photographic picture.

Fixed PC12 cells were treated with PS or PC at their CMCs of 2 and 90 mM, respectively, for 30 min. The fixed cells were washed with deionized water to remove excess salts and were then dried by N_2_ gas flow in preparation for KPFM measurement.

### AFM and KPFM scanning

**Figure 1:**
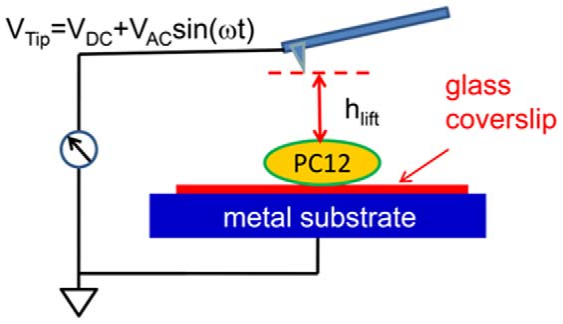
Schematic representation of the experimental setup to measure the Φ_s_ of a fixed PC12 cell. During the KPFM scan, the tip was lifted a height of 30 nm and applied with an alternative-current voltage (V_AC_ sin (ωt)) and a direct-current voltage (V_DC_), and the substrate was connected to the ground.

The KPFM measurements were performed by AFM (Digital Instruments/Veeco Bioscope SZ) as reported in previous studies [20]. Conductive AFM tips (PPP-EFM, Nanosensors) were used, and scanning was performed in lift mode based on a two-pass scan, including one trace and its retrace (Fig. 1). In the first pass, a typical topological AFM image was taken in tapping mode with a zero tip bias on the trace and retrace. In the second pass, the AFM tip was lifted to 30 nm above the sample surface (h_lift_) with an applied tip bias voltage (V_Tip_) to measure the Φ_s_. Coverslip containing the fixed cells was placed on a metal substrate and set to electrical ground by connecting it to the sample bias control of the scanning probe microscope (SPM) controller. An alternating current voltage (V_AC_) combined with a direct current voltage (V_DC_) were applied to the tip during the scan in lift mode; that is, V_Tip_ = V_DC_+V_AC_ sin (ωt). With V_AC_ = 6 V, the cantilever oscillated at its natural resonance frequency of ω (∼70 kHz). Therefore, the voltage between tip and sample is ΔV = Δφ−V_DC_+V_AC_ sin (ωt), where Δφ is the contact potential difference.

The conductive tip and sample were extremely close and may be regarded as a capacitor with the energy 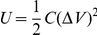, where C is the capacitance between the tip and the sample. The electrostatic force between the tip and the sample with a distance z can be calculated by 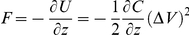. By inserting 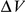, the force can be divided into three components: DC (F_DC_), frequency of ω (F_ω_), and frequency of 2ω (F_2ω_) terms. Using the electronic system of a lock-in technique, 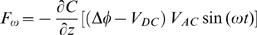 can be singled out. Therefore, the tip will oscillate at frequency ω if the tip is scanning in non-contact mode. When the tip oscillation vanishes, the applied V_DC_ is equal to the contact potential difference Δφ. Thus the 2D work function image (ie surface potential image for non-metallic material) was obtained through the feedback circuit of the electronic system of KPFM. This oscillation was detected by an SPM photodiode, which then sent a KPFM signal (as feedback) through a lock-in amplifier built in the SPM controller to isolate the oscillation mode at ω. The given V_DC_, applied to the conductive tip, was regulated by a feedback loop to adjust a minimal electrostatic interaction between the tip and the sample [16], [21]. A highly oriented pyrolytic graphite surface (ZYB grade) (Momentive Performance Materials, OH, USA), with a work function of approximately 4.65 eV, was used as a reference to calibrate the measured Φ_s_.

### Flow cytometer

The PC12 cells (∼10^5^) were washed three times with PBS and suspended in a trypsin-EDTA solution (0.05% trypsin and 0.5 mM EDTA in PBS). The cell suspension was centrifuged at 800× g for 5 min and the pellet was resuspended with 100 µl of binding buffer (10 mM HEPES, 140 mM NaCl, and 2.5 mM CaCl_2_; pH 7.4). The buffer containing the cells was added with annexin V-fluorescein isothiocyanate (5 µl) and stayed in darkness for 5 min. Thereafter, 5 µl of 50 µg/ml propidium iodide (PI) was added 1 min before the cells was placed into the flow cytometer (FACSCanto II, BD, NJ, USA). The annexin V-fluorescein isothiocyanate (FITC)-PI assay kit was purchased from BioVision, Inc (CA, USA). PI is a membrane-impermeable DNA binding fluorescent molecule, and a high level of PI fluorescence indicates damage in the intactness of the membrane.

### Data Analysis

Membrane patches of 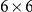 µm^2^ were scanned by KPFM at a resolution of 256
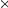
128 pixels. All Φ_s_ values for a scanned patch were binned at 40 steps, according to the maximal and minimal Φ_s_ values (usually with a difference less than 0.15 V) obtained in that patch. The histogram curve was fitted with a two-peak Gaussian model: 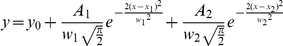; where y_0_ represents baseline offset, A1 & A2 are areas under the two Gaussian curves from the baseline, *x*_1_ and *x*_2_ are centers of the two Gaussian peaks, and *w_1_* and *w_2_* are 2×σ (approximately 0.849, the width of the perspective peak at half of the height).

To characterize the distribution profile of Φ_s_s relative to the mean value, the skewness of the histogram of each KPFM image was defined as: 
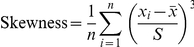
, where *n* is number of pixels in a KPFM image, 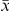 is the mean of the surface potentials obtained by KPFM, and *S* is the standard deviation of the data. A negative skewness value indicates that the tail of the asymmetric histogram is longer at the left of the peak than at the right, and a positive value indicates a longer tail at the right side of the peak. Data are presented as mean ± standard error of the mean (SEM).

## Results and Discussion

**Figure 2:**
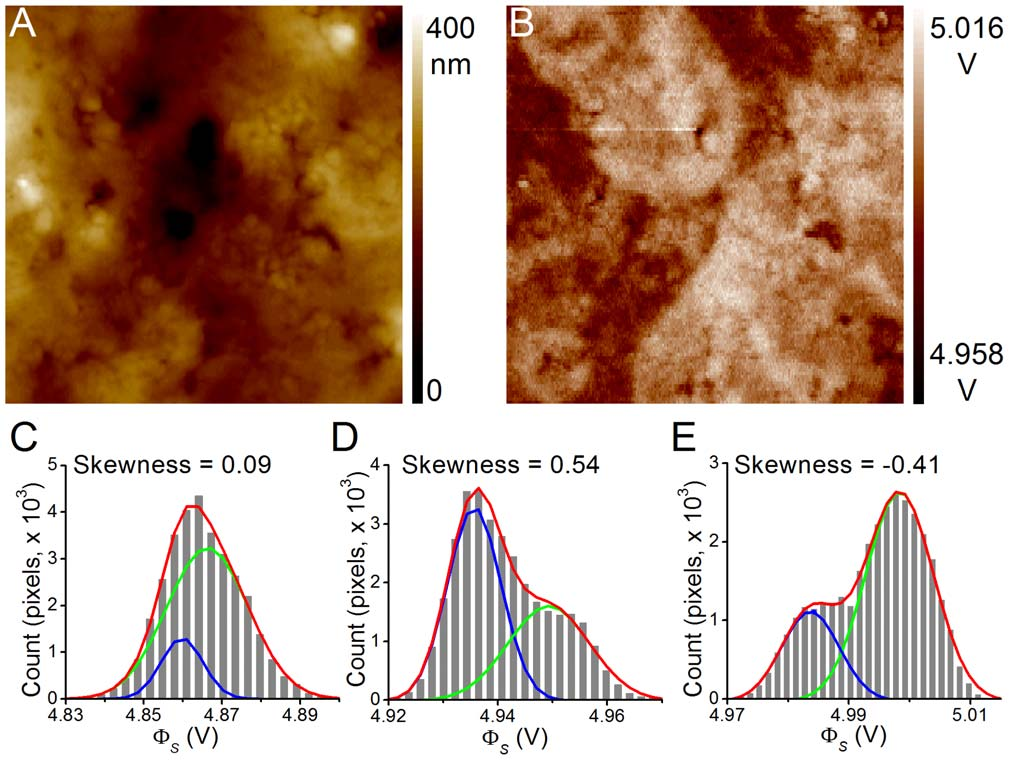
Various Φ_s_ distribution histograms of PC12 cell plasma membrane. A fixed PC12 cell was air-dried and then a 6
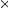
6 µm^2^ region of the plasma membrane was scanned at a resolution of 256
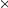
128 pixels. Figures A and B show the simultaneously recorded AFM and KPFM images, respectively. C. The Φ_s_ from B was binned with 40 steps and the histogram was fitted with a two-peak Gaussian model (red line). The two Gaussian peaks were plotted in green and blue. D–E. Two other representative cells having Φ_s_ distribution histograms with differing skewness values.

The extracellular surface of plasma membrane contains various charged phospholipids, proteins, and polysaccharides which form an electrostatic layer. To characterize the Φ_s_ of the plasma membrane, fixed PC12 cells were dried in air and a 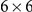 µm^2^ region on the cell surface was scanned in both AFM and KPFM modes (Figs. 2A and B, respectively). The AFM images revealed that the membrane surfaces were not flat, and the KPFM images confirmed that Φ_s_ was not homogenously distributed on the membrane surface. However, we found no direct relationship between surface height (obtained by AFM) and Φ_s_ (scanned by KPFM). The measured Φ_s_ values were ∼5 V, and the difference between the maximum and minimum Φ_s_ in each scanned membrane patch was ∼0.15 V. To characterize the Φ_s_ distribution, all Φ_s_ values in a scanned patch were binned at 40 steps and most of the histograms showed an asymmetrical distribution pattern. To characterize the asymmetry, the skewness value was calculated and three types of distribution patterns were identified (Figs. 2C–E). Some histograms were symmetric with a skewness value close to zero (Fig. 2C), and others had a longer tail either to the right (positive skewness, Fig. 2D) or left (negative skewness, Fig. 2E). Out of all 78 cells that were scanned, 49 obtained a negative value for skewness, indicating the various Φ_s_ distribution patterns in different cells.

To characterize whether a composition change in the outer membrane leaflet would affect the Φ_s_ distribution profile, fixed PC12 cells were incubated in solutions containing negatively charged PS- or neutral PC-micelles for 30 min. The incorporation of PS onto the outer membrane leaflet was verified by annexin V staining (Supporting Figure S2). Annexin V has a specific affinity with PS. The intensity of the conjugated fluorescence molecule increased in cells treated with PS-micelles but not in cells treated with PC-micelles.

**Figure 3:**
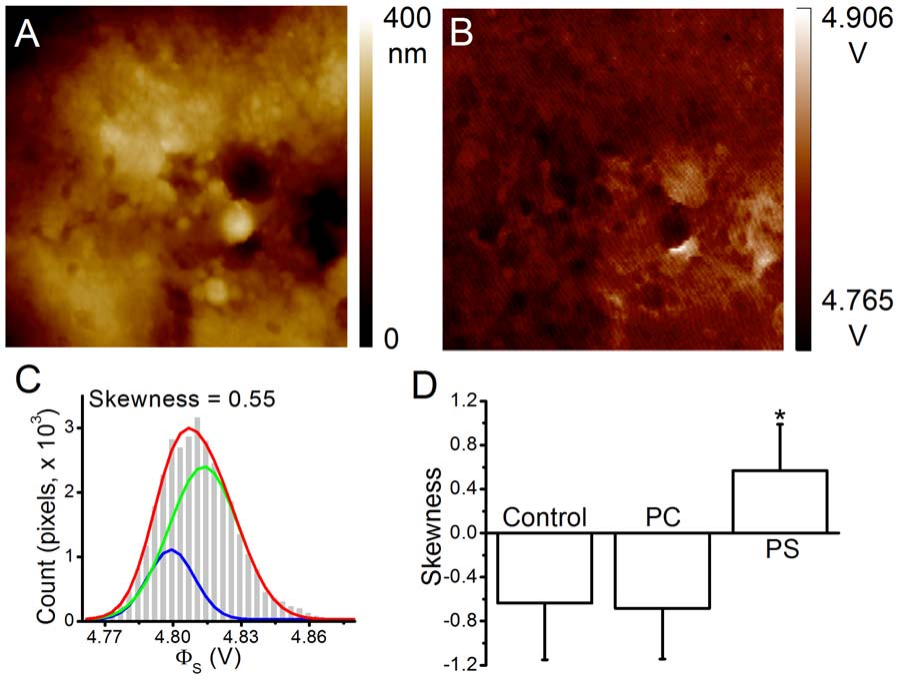
PS-micelles altered the Φ_s_ distribution histogram. Figures A and B show the AFM and KPFM images, respectively, of a PC12 cell treated with PS-micelles. C. The Φ_s_ from B was binned with 40 steps and the histogram was fitted with a two-peak Gaussian model (red line). The two Gaussian distributions were plotted in green and blue. D. Averaged skewness in PC12 cells without treatment (control) or treated with PC- and PS-micelles (PC & PS, respectively). Data are expressed as mean ± SEM, and N = 11 for each group. * indicates *p*<0.05 (by Student's *t*-test) compared with the control group.

After the micelles had been incorporated into the fixed PC12 cells, the unbound micelles were washed off and the cells were air-dried for KPFM scanning. Figs. 3A and B show the AFM and KPFM images, respectively, of a cell treated with PS-micelles. The AFM image did not show a significant accumulation of particles at the cell surface. The overall Φ_s_ was ∼5 V which was similar to those of other cells; however, the Φ_s_ distribution histogram of this PS-micelle-treated PC12 cell had a skewness value of 0.55 (Fig. 3C). The averaged results showed that the skewness for the control (n = 12) and PC-micelle-treated (n = 12) cells were 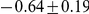 and 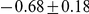, respectively. The skewness was significantly altered in cells treated with PS-micelles, to 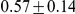. It is possible that the amount of PS-micelle incorporation was not large enough to vary the overall Φ_s_; however, the distribution histogram pattern was still altered. These results revealed that the incorporation of PS-micelles onto the plasma membrane outer leaflet can be monitored by KPFM, with changes being evident in the Φ_s_ distribution histogram.

Parkinson's disease (PD) is a movement disorder in humans caused by the progressive degeneration of dopaminergic neurons in the nigrostriatal pathway [22]. Dopamine, an oxidizable chemical that is stored in dopaminergic neurons, might be related to the neurodegenerative PD [23]. In addition, a pathological factor identified in PD patients is a higher than normal concentration of Zn^2+^ in the substantial nigra region [24].

**Figure 4:**
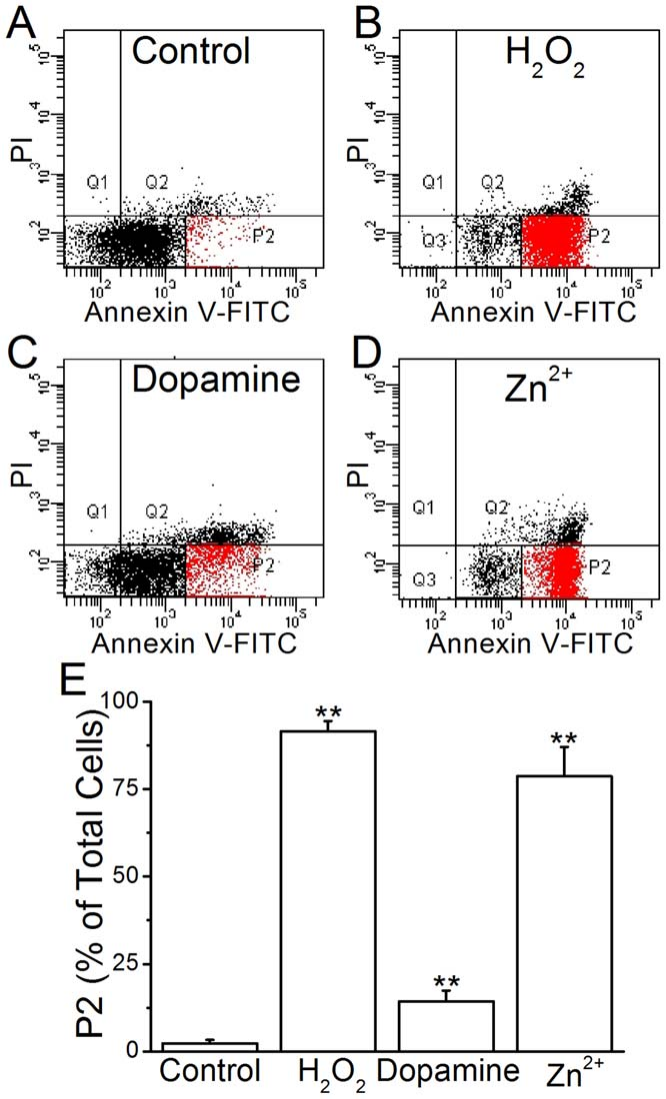
Chemical treatments increased the surface PS content. Figures A–D. The distribution plots of PC12 cells at various PI and annexin V fluorescence intensities, after treatment with different chemicals. Q1–Q4 represent four quadrants with various levels of PI and annexin V fluorescence intensities. E. The averaged P2 fraction across all cells. The P2 fraction (red) was the cells distributed in Q4 quadrant with FITC intensity over 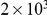. The data represent mean
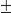
SEM from three batches of cells. ** indicates *p*<0.01 (by Student's *t*-test) compared with the control group.

The presence of negatively charged PS on the membrane outer surface is an important signal for apoptosis [25]. To determine whether dopamine and Zn^2+^ would induce the translocation of PS to the outer membrane leaflet, FITC-conjugated annexin V was used to label the exposed PS and the results were analyzed by flow cytometry. Annexin V is a protein with a molecular weight of ∼40 kDa, which cannot diffuse freely through the plasma membrane. Thus if a plasma membrane is intact, only the exposed PS will be bound by annexin V. The membrane-impermeable fluorescent DNA marker PI was applied to verify the intactness of the cells. H_2_O_2_, an oxygen-releasing reagent, has been widely used to apply oxidative stress on cells and induce apoptosis [26]. Figs. 4A–D show the fluorescence intensity distributions of cells stained with FITC-annexin V and PI. The average P2 fractions (FITC fluorescence intensity above 2000 and PI fluorescence below 200 arbitrary units) across all cells were analyzed and the results are shown in Fig. 4E. The low PI fluorescence level indicated that cells were alive and exhibited no membrane leakage. The proportion of this fraction increased significantly from 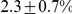 in control cells to 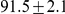, 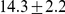, and 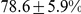 in cells pretreated with H_2_O_2_, dopamine, and Zn^2+^, respectively. The results suggested that these chemical treatments caused the exposure of PS to the outer membrane leaflet.

**Figure 5:**
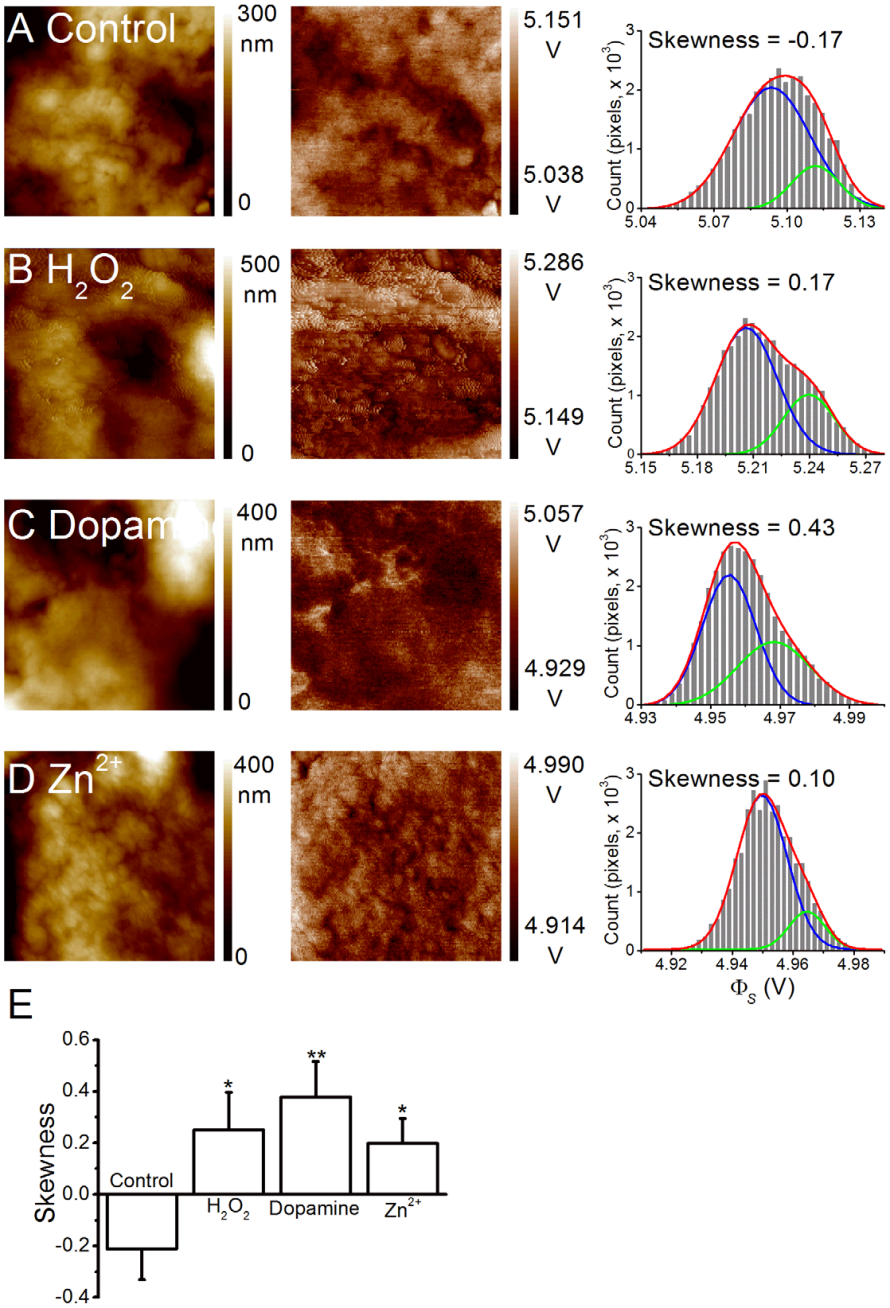
Oxidative stresses shifted the skewness to a positive value. Figures A–D. are the AFM (left column) and KPFM (middle column) images from representative PC12 cells treated with plain buffer (Control), H_2_O_2_, dopamine, or Zn^2+^, respectively. The Φ_s_ values were binned with 40 steps, and the skewness value of each cell was analyzed (right column). The distribution was fitted with a two-peak Gaussian model (red line) and the two Gaussian curves were plotted in blue and green. E. Plots of the average skewness values of cells with various treatments. The sample numbers for groups A to D were 42, 14, 29, and 28, respectively. * and ** indicate *p*<0.05 and *p*<0.01, respectively, compared with the control group (by Student's *t*-test).

To further verify whether changes in the membrane surface environment were detected by KPFM, fixed PC12 cells that had been treated with H_2_O_2_, dopamine, or Zn^2+^ were scanned in AFM and KPFM modes. Figs. 5A–D show the representative AFM and KPFM images from cells treated with each chemical and the histograms of Φ_s_ distribution from the same representative cells. The distribution histograms for treated cells were noticeably altered. The average skewness of the Φ_s_ histogram for control cells was 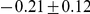 (n = 42), and the value was significantly increased to 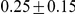 (n = 14), 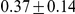 (n = 29), and 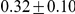 (n = 28) for cells treated with H_2_O_2_, dopamine, and Zn^2+^, respectively (Fig. 5E). These results suggested that changes in the charge on a membrane surface due to various stimulants could be verified by KPFM.

The biomolecules on a cell surface are complicated and contribute differentially to the Φ_s_. As revealed by electrostatic force microscopy, the electrostatic potentials on the surface of the biofilms formed by bacteria differ according to differing nutrient formulas [27]. In addition, the zeta potential of E. coli is changed by the application of an antimicrobial peptide [28]. Previous research has also applied AFM to investigate the effective surface charge of a mixture of PS and PC [14]. However, to date no reports had been published about the electrostatic properties of native membrane at high resolution.

KPFM measures the potential difference between two surfaces at close proximity. The main contribution of this potential difference is the electric charge. Paraformaldehyde fixation forms methylene bridges between molecules and dose not generate or eliminate electric charge; therefore, fixation reaction does not likely to affect the KPFM measurement. Considering the fluctuation in the surface heights of native plasma membrane, the current study adopted a 30 nm lift for the scanning probe. This setting allowed us to obtain both AFM and KPFM images in good resolution over a broad spatial range, without contamination by the plasma membrane. Although KPFM cannot operate properly in aqueous conditions, a previous study reported that the Φ_s_s of the dried protein/DNA nanoarray measured by KPFM correlated with the isoelectric point of the bound molecule [29]. Our previous study measuring the potentials of biomolecules modified on a nanowire under a dried condition yielded results that corresponded closely with those of other approaches carried out in a liquid phase [20]. Therefore, the membrane Φ_s_s measured in a dried state correlate with its electrostatic properties in solution.

The averaged Φ_s_s of the measured PC12 cells were approximately 5 V in each case, and did not differ significantly among the treatment groups. However, the distribution histogram of the Φ_s_s in each cell usually shows an asymmetrical pattern, which can be represented by the skewness value [30]. The PC12 cells displayed various levels of PS exposure at the outer membrane leaflet, as revealed by annexin V staining, but only a small portion (5.2%) of the cells showed a high level of staining (Supporting Information S2A). After the oxidative stress treatments, the PS exposure levels were significantly elevated. The translocation of PS from the inner to outer leaflet, or the incorporation of PS micelles into the membrane, did not change the overall averaged Φ_s_s but did result in a positive shift in the skewness value. In addition, other surface modifications such as polysaccharides can change the anionic charge level in response to various physiological activities [7], [9]. In short, all of these factors contribute to the asymmetry distribution pattern. Nevertheless, the mechanism by which these changes correlate with the skewness value needs further investigation.

The exposure of PS from the inner to outer plasma membrane leaflet is an early signal of programmed cell death [31]. Dopamine treatment shifts the skewness to a positive value in a similar manner to that induced by Zn^2+^ and H_2_O_2_ treatment, although dopamine only slightly (but significantly) increases the PS exposure level. Therefore, it is possible that dopamine, H_2_O_2_, and Zn^2+^ are mediated by different pathways to induce cell death [32], [33]. Furthermore, the findings demonstrate the involvement of various molecules at the plasma membrane, in addition to PS exposure, which result in changes to the Φ_s_s distribution profile.

### Conclusion

To the best of our knowledge, this was the first study to report on the use of KPFM to characterize the native Φ_s_ of biological plasma membranes under oxidative stress. KPFM has been applied to measure the potentials of avidin and DNA at molecular level [34]; the negative potential of a single strand of DNA can be masked by the interaction with its complementary DNA strand [29]. Our results revealed that Φ_s_ distribution histograms were altered by PS micelle incorporation and various ROS stimulations. It is not clear how the compositions of charged molecules at the membrane surface are altered to induce these changes in the Φ_s_ distribution histogram. By applying treatments against specific molecules at the membrane surface, we were able to characterize the correlation of charged molecules with Φ_s_. Moreover, in conjunction with other indicators such as lipid raft fluorescence dyes, KPFM can be applied to characterize the Φ_s_ changes at specific regions. This study demonstrated that KPFM can act as a biosensor to characterize the structural and electrical properties of biological samples, with changes being detected that reflect the physiological conditions of cells.

## Supporting Information

Figure S1 Critical micelle concentrations of PC and PS. The surface tension of PC and PS were measured by pendant drop profile analysis tensiometry to determine the CMCs. A. Schematic illustration of the reversible monomer-micelle equilibrium. The surfactant head is hydrophilic, and the surfactant tail is hydrophobic. When surfactant molecules are dissolved in water at concentrations above the CMC, micelles will be formed. B–C. Surface tensions of PS- and PC-containing solutions at various concentrations. The CMCs of PS and PC were determined at 1.8 and 80 mM, respectively.
(TIF)

Figure S2 Incorporation of micelles onto PC12 plasma membrane. The incorporation of PS micelles onto the plasmam membrane of PC12 cells was verified by the binding of FITC-conugatted annexin V. Fixed PC12 cells were incubated in PBS buffer containing PC (90 mM) or PS (2 mM) for 30 min and then stained with PI and FITC-conjugated annexin V. The fluorescence intensities of PI and annexin V-FITC of each cell were measured by flow cytometry. A–C. The distributions of PC12 cells with different PI and FITC fluorescence intensities are plotted after treatment with buffer only (Control) or buffer containing PC- or PS-micelles. Q1–Q4 are four quadrants with various levels of PI and FITC fluorescence intensities. The proportions of the cells with FITC fluorescence intensity over 2000 (arbitrary units) and PI staining signal below 200 (arbitrary units) were 5.2 and 2.3% for the control and the cells treated with PC-micelles, respectively. This proportion was dramatically elevated to 73.1% in the cells treated with PS-micelles.
(TIF)

## References

[1] Garcia-Saez AJ, Schwille P (2010). Stability of lipid domains.. FEBS Lett.

[2] Subczynski WK, Wisniewska A (2000). Physical properties of lipid bilayer membranes: relevance to membrane biological functions.. Acta Biochim Pol.

[3] Leventis PA, Grinstein S (2010). The distribution and function of phosphatidylserine in cellular membranes.. Annu Rev Biophys.

[4] Varki A (2011). Evolutionary forces shaping the Golgi glycosylation machinery: why cell surface glycans are universal to living cells.. Cold Spring Harb Perspect Biol.

[5] Janas T (2011). Membrane oligo- and polysialic acids.. Biochim Biophys Acta.

[6] Li CX, Jing YL, Xie YK (2007). Glycosylation-induced depolarization facilitates subthreshold membrane oscillation in injured primary sensory neurons.. Brain Res.

[7] Shatnyeva OM, Kubarenko AV, Weber CE, Pappa A, Schwartz-Albiez R (2011). Modulation of the CD95-induced apoptosis: the role of CD95 N-glycosylation.. PLoS One.

[8] Dall'olio F, Malagolini N, Trinchera M, Chiricolo M (2012). Mechanisms of cancer-associated glycosylation changes.. Front Biosci.

[9] Kontou M, Weidemann W, Bork K, Horstkorte R (2009). Beyond glycosylation: sialic acid precursors act as signaling molecules and are involved in cellular control of differentiation of PC12 cells.. Biol Chem.

[10] Engel A, Muller DJ (2000). Observing single biomolecules at work with the atomic force microscope.. Nature Structural Biology.

[11] Tsai CC, Lin CL, Wang TL, Chou AC, Chou MY (2009). Dynasore inhibits rapid endocytosis in bovine chromaffin cells.. American Journal of Physiology - Cell Physiology.

[12] Fisher TE, Marszalek PE, Fernandez JM (2000). Stretching single molecules into novel conformations using the atomic force microscope.. Nature Structural Biology.

[13] Marshall BT, Long M, Piper JW, Yago T, McEver RP (2003). Direct observation of catch bonds involving cell-adhesion molecules.. Nature.

[14] Yang Y, Mayer KM, Hafner JH (2007). Quantitative membrane electrostatics with the atomic force microscope.. Biophys J.

[15] Kalinin SV, Rodriguez BJ, Jesse S, Karapetian E, Mirman B (2007). Nanoscale Electromechanics of Ferroelectric and Biological Systems: A New Dimension in Scanning Probe Microscopy.. Annual Review of Materials Research.

[16] Palermo V, Palma M, Samori P (2006). Electronic characterization of organic thin films by Kelvin probe force microscopy.. Advanced Materials.

[17] Allison DP, Mortensen NP, Sullivan CJ, Doktycz MJ (2010). Atomic force microscopy of biological samples.. Wiley Interdiscip Rev Nanomed Nanobiotechnol.

[18] Lee JD, Huang PC, Lin YC, Kao LS, Huang CC (2008). In-depth fluorescence lifetime imaging analysis revealing SNAP25A-Rabphilin 3A interactions.. Microsc Microanal.

[19] McGee R (1980). Regulation of presynaptic cellular function. Biochemical studies using clonal neuronal cells.. Mol Cell Biochem.

[20] Tsai C-C, Chiang P-L, Sun C-J, Lin T-W, Tsai M-H (2011). Surface Potential Vairations on a Silicon Nanowire Transistor in Biomolecular Modification and Detection.. Nanotechnology.

[21] Fujihira M (1999). Kelvin probe force microscopy of molecular surfaces.. Annual Review of Materials Science.

[22] Lo HS, Chiang HC, Lin AMY, Chiang HY, Chu YC (2004). Synergistic effects of dopamine and Zn^2+^ on the induction of PC12 cell death and dopamine depletion in the striatum: possible implication in the pathogenesis of Parkinson's disease.. Neurobiology of Disease.

[23] Leong SL, Cappai R, Barnham KJ, Pham CL (2009). Modulation of alpha-synuclein aggregation by dopamine: a review.. Neurochem Res.

[24] Barnham KJ, Bush AI (2008). Metals in Alzheimer's and Parkinson's diseases.. Curr Opin Chem Biol.

[25] Schlegel RA, Williamson P (2001). Phosphatidylserine, a death knell.. Cell Death and Differentiation.

[26] Konat GW (2003). H_2_O_2_-induced higher order chromatin degradation: a novel mechanism of oxidative genotoxicity.. J Biosci.

[27] Oh YJ, Jo W, Yang Y, Park S (2007). Biofilm formation and local electrostatic force characteristics of Escherichia coli O157:H7 observed by electrostatic force microscopy.. Appl Phys Lett.

[28] Alves CS, Melo MN, Franquelim HG, Ferre R, Planas M (2010). Escherichia coli cell surface perturbation and disruption induced by antimicrobial peptides, BP100 and pepR.. J Biol Chem.

[29] Sinensky AK, Belcher AM (2007). Label-free and high-resolution protein/DNA nanoarray analysis using Kelvin probe force microscopy.. Nature Nanotechnology.

[30] Altman DG, Bland JM (1996). Detecting skewness from summary information.. Bmj.

[31] Fadok VA, Voelker DR, Campbell PA, Cohen JJ, Bratton DL (1992). Exposure of phosphotidylserine on the surface of apoptotic lymphocytes triggers specific recognition and removal by macrophages.. Journal of Immunology.

[32] Ahmadi FA, Grammatopoulos TN, Poczobutt AM, Jones SM, Snell LD (2008). Dopamine selectively sensitizes dopaminergic neurons to rotenone-induced apoptosis.. Neurochem Res.

[33] Garai K, Sahoo B, Kaushalya SK, Desai R, Maiti S (2007). Zinc lowers amyloid-beta toxicity by selectively precipitating aggregation intermediates.. Biochemistry.

[34] Leung C, Kinns H, Hoogenboom BW, Howorka S, Mesquida P (2009). Imaging surface charges of individual biomolecules.. Nano Lett.

