# Supplementary figures and images for: Changes in Plasma Membrane Surface Potential of PC12 Cells as Measured by Kelvin Probe Force Microscopy
Source: PLoS One. 2012 Apr 10;7(4):e33849. doi: 10.1371/journal.pone.0033849 (PMC3323603; doi:10.1371/journal.pone.0033849)

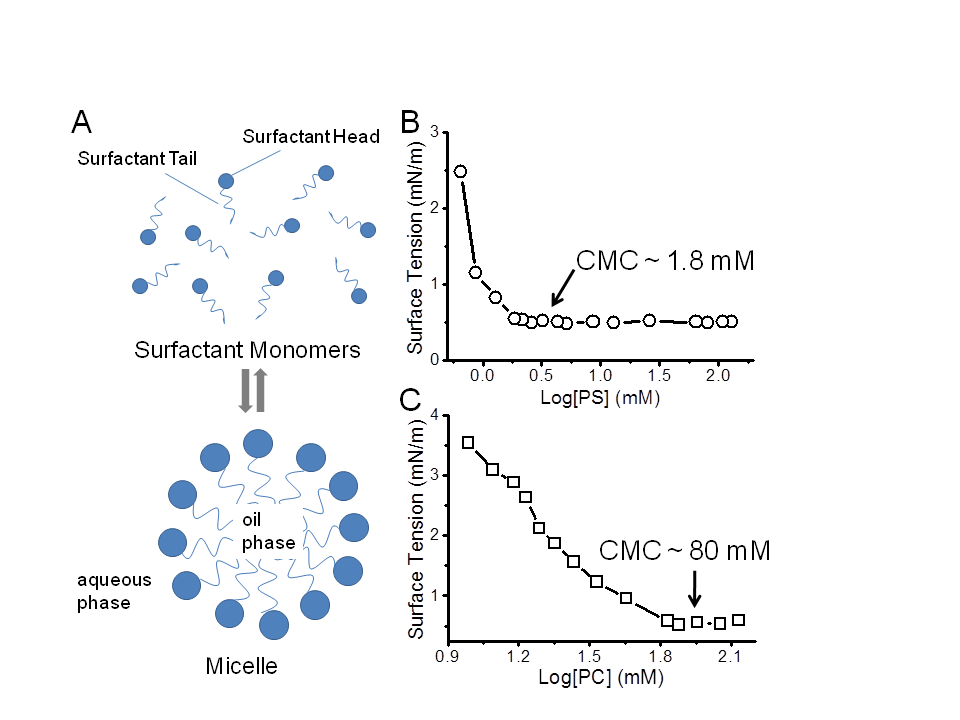

Supplement: Figure S1 — Critical micelle concentrations of PC and PS. The surface tension of PC and PS were measured by pendant drop profile analysis tensiometry to determine the CMCs. A. Schematic illustration of the reversible monomer-micelle equilibrium. The surfactant head is hydrophilic, and the surfactant tail is hydrophobic. When surfactant molecules are dissolved in water at concentrations above the CMC, micelles will be formed. B–C. Surface tensions of PS- and PC-containing solutions at various concentrations. The CMCs of PS and PC were determined at 1.8 and 80 mM, respectively. (TIF) [file pone.0033849.s001.tif]
